# The role of IGF1 in determining body composition in children and adolescents with growth hormone deficiency and those with idiopathic short stature

**DOI:** 10.1007/s12020-024-03992-0

**Published:** 2024-08-14

**Authors:** Hussein Zaitoon, Michal Yackobovitch-Gavan, Eyas Midlej, Adi Uretzky, Irina Laurian, Anna Dorfman, Hagar Interator, Yael Lebenthal, Avivit Brener

**Affiliations:** 1 The Institute of Pediatric Endocrinology, Diabetes and Metabolism, Dana-Dwek Children’s Hospital, Tel Aviv Sourasky Medical Center, Tel Aviv, Israel; 2 Faculty of Medicine, Tel Aviv University, Tel Aviv, Israel; 3 Department of Epidemiology and Preventive Medicine, School of Public Health, Faculty of Medicine, Tel Aviv University, Tel Aviv, Israel

**Keywords:** bioelectrical impedance analysis (BIA), fat percentage, muscle mass, muscle-to-fat ratio, pediatric patients, sex dimorphism

## Abstract

**Purpose:**

Treatment with recombinant human growth hormone (rhGH) increases insulin growth factor-1 (IGF1) levels, therefore, monitoring both IGF1 and growth constitutes an acceptable parameter of therapeutic safety and efficacy. We aimed to investigate the relationship between IGF1 level and body composition in children and adolescents undergoing rhGH therapy for growth hormone deficiency (GHD) and idiopathic short stature (ISS).

**Methods:**

This observational retrospective study included the bioimpedance analysis (BIA) reports (*n* = 305) of 135 pediatric patients (age 5–18 years), 64 with GHD and 71 with ISS, conducted as part of routine clinic visits. Sociodemographic and clinical data were extracted from medical records. Generalized estimating equations linear models were used to explore the contributing factors for body composition components of fat percentage (FATP), appendicular skeletal muscle mass (ASMM) z-score, and muscle-to-fat ratio (MFR) z-score while adjusting for cumulative doses of rhGH.

**Results:**

Subjects with GHD exhibited higher body mass index z-scores (*p* < 0.001), higher FATP and truncal FATP scores, lower MFR z-score, and higher diastolic blood pressure percentiles than the ISS group (*p* = 0.010, *p* = 0.027, *p* = 0.050, and *p* = 0.050, respectively). Female sex (*p* < 0.001) and a GHD diagnosis (*p* < 0.001), were major contributors to higher FATP scores; female sex (*p* = 0.049) and ISS diagnosis (*p* = 0.005) were major contributors to higher MFR z-scores; and female sex (*p* < 0.001), older age (*p* < 0.001) and higher insulin-like growth factor 1 z-scores (*p* = 0.021) were major contributors to higher ASMM z-scores. Socioeconomic position and cumulative rhGH dose were not significant contributors to body composition parameters.

**Conclusion:**

Children with GHD, including those undergoing rhGH treatment, may be at risk for increased adiposity and associated metabolic implications. Sex- and age-adjusted IGF1 levels were related to muscle mass but not to adiposity. Hence, rhGH treatment aimed at increasing IGF1 levels may alleviate these effects by promoting muscle growth.

## Introduction

Short stature, defined as height at or below two standard deviations below the mean for sex and age, is a common reason for referral to pediatric endocrinologists. The initial assessment entails ruling out any underlying medical conditions or genetic syndromes that could hinder normal growth. This is frequently followed by the assessment of growth hormone (GH) secretion and its efficacy as measured by insulin-like growth factor 1 (IGF1) levels, as well as by conducting GH stimulation tests. The past decade has witnessed the expansion of the prescription of recombinant human growth hormone (rhGH) to a broader range of indications and greater usage beyond simply replacing growth hormone deficiency (GHD) to encompass hormonal augmentation therapy in situations where deficiency has not been established and idiopathic short stature (ISS) is defined [1].

GH is an anabolic hormone that determines lean body mass. It stimulates whole-body anabolism with protein accretion occurring primarily in skeletal muscle, while also exerting tissue-specific effects on other body tissues. IGF1 is synthesized within the liver and released into the bloodstream in response to GH stimulation, with its concentrations serving as an indicator of GH status [2]. In addition to its impact on increasing linear growth, IGF1 facilitates muscle growth through various pathways [3, 4].

The secretion of IGF1 and its activity can be influenced by nutritional status in young children with undernutrition-depressing IGF1 production [5]. Furthermore, food deprivation may dampen the myogenic effects of IGF1 stimulation, as demonstrated in animal models [6]. Treatment with rhGH increases IGF1 levels, therefore, monitoring both IGF1 and growth constitutes an acceptable parameter of therapeutic safety and efficacy [7, 8]. However, there is a lack of data regarding the impact of rhGH treatment on body composition in relation to IGF1 levels.

In January 2018, our Pediatric Endocrine Unit implemented the analysis of body composition by means of bioimpedance analysis (BIA) as part of the standard intake assessment of subjects referred for endocrine consultation [9]. The escalating rates of obesity have led to a significant upsurge in cardiovascular risk factors in youth [10–12]. In addition, low muscle mass may increase the risk for cardiovascular complications, even in the absence of obesity [13]. While the body mass index (BMI) percentile is the most common tool used for identifying children at risk, it lacks the capacity to identify the ones with low muscle mass whose BMI percentiles may be within the normal range. Our group described the role of body composition assessment in identifying cardiovascular risk factors in pediatric patients with overweight/obesity, celiac disease, and non-classic congenital adrenal hyperplasia [14–16]. In the current study, we investigated the role of IGF1 levels in the determination of body composition components in children and adolescents treated with rhGH for short stature.

## Methods

### Study design and population

This real-life, observational study included pediatric participants (aged 5–18 years) followed for either GHD or ISS at the Institute of Pediatric Endocrinology in Dana-Dwek Children’s Hospital. Each patient underwent at least one body composition assessment BIA during routine follow-up between January 2018 and August 2023.

Querying the BIA database yielded a list of patients with the sole diagnosis of either GHD or ISS. The electronic medical records of those children and adolescents were examined, and sociodemographic and clinical data at the time of BIA assessment were retrieved. Excluded were patients with genetic syndromes, multiple pituitary hormone deficiency, a history of malignancy treated with chemotherapy and/or radiotherapy, and medical conditions and/or medications that could adversely affect growth. Sociodemographic characteristics, perinatal history, medical conditions, medications for, and family history of cardiovascular disease risk factors (obesity, hypertension, type 2 diabetes, and dyslipidemia) were retrieved from the hospital’s electronic medical files. Clinical data, anthropometric measurements, vital signs, pubertal staging, and laboratory evaluation were extracted at the time of BIA assessments. The study protocol was approved by our medical center’s Institutional Review Board which waived informed parental consent (065-18-TLV). The data were handled in accordance with the principles of good clinical practice.

### Definitions of study variables

#### Socioeconomic position (SEP) grading

The SEP as determined by home address (SEP cluster and SEP index) was based upon the Israel Central Bureau of Statistics’ Characterization and Classification of Statistical Areas within Municipalities and Local Councils by Socio-Economic Level of the Population 2015 [17].

#### Growth hormone axis evaluation

Patients were referred for a GH provocation test after completing laboratory tests to exclude chronic medical conditions, such as hypothyroidism, active celiac disease, chronic renal insufficiency, and inflammatory bowel disease. Patients with a peak GH level lower than 7.5 ng/mL were referred for a second provocation test. Sex hormone priming of testosterone or estradiol was administered prior to testing in boys older than 13 years and girls older than 11 years, respectively [18]. The diagnosis of isolated GHD required evidence of insufficient GH reserve in two stimulation tests performed with different stimuli (glucagon, clonidine, and arginine) and with no evidence of other hypothalamic-pituitary dysfunction [19]. Patients diagnosed with GHD were referred for brain magnetic resonance imaging (MRI) to assess the structure of the pituitary gland and rule out any underlying structural abnormalities or lesions that could impact the management of GHD before the initiation of rhGH therapy.

#### rhGH therapy

Definitions of diagnoses and therapeutic doses of rhGH according to indication have evolved since the original FDA indication for GHD in 1985 [18]. In 2003, the FDA expanded rhGH use to the treatment of ISS, defined as height z-score ≤−2.25 in a healthy child with sufficient GH reserve [18]. Of note, the EMEA has not granted approval for this indication, resulting in limited global adoption. Nevertheless, the Israeli Ministry of Health has granted approval for it, following the guidelines published in 2016 [8]. The decision for rhGH treatment of children and adolescents diagnosed as having ISS was made jointly by the patient, the parents, and the treating pediatric endocrinologist. The initial dosage was determined according to the 2016 guidelines with subsequent adjustments made according to the findings of clinical and laboratory assessments. The rhGH dosage was recorded during the BIA assessment, and the total cumulative rhGH dosage was determined by multiplying the recommended average daily dose (in μg/kg/day) by the total number of days the treatment had been administered, with the final result being converted from micrograms to grams.

#### IGF1 monitoring

The goal of rhGH treatment is to promote linear growth and help individuals achieve a normal height for their sex and age. The dosage is adjusted according to the individual’s response to treatment and other clinical considerations, such as growth velocity and pubertal stage [1]. Adherence and IGF1 production in response to rhGH dose changes is monitored by periodic measurement of serum IGF1 levels. Furthermore, the interpretation of IGF1 levels with respect to sex, age, and pubertal development stages serves to aid in the prevention of hyper-augmentation of the GH-IGF1 axis, with the goal of avoiding an IGF1 exceeding two z-scores [8]. In the current study, IGF1 levels were converted to sex- and age-appropriate z-scores according to normal reference ranges in order to allow standardization between individuals [20, 21].

#### Clinical characteristics

Height, weight, and BMI calculated as weight (kg) divided by height (m) squared were converted to sex- and age-specific standard deviation scores (z-scores) by means of CDC 2000 growth charts [22]. The routine clinical evaluation in the endocrine clinic also included the calculation of mid-parental height (paternal height [cm] + maternal height [cm] ± 13 cm/2), from which the mid-parental height z-scores were derived [23]. Delta height z-scores were calculated as the difference between the patient’s current height z-score and the mid-parental height z-score. Weight status was defined according to BMI z-scores as follows: underweight as BMI percentile ≤5th percentile (z-score ≤−1.645), overweight as BMI percentile ≥85th and <95th percentiles (1.04 ≤ z-score <1.645), and obesity as BMI percentile ≥95th percentile (z-score ≥1.645) [24, 25].

Prematurity was defined as gestational age (GA) < 37 weeks. Birth weight z-scores were calculated by PediTools Electronic Growth Chart Calculators based on the Fenton growth chart for preterm infants [26]. Appropriate birth weight for gestational age (AGA) was defined as corrected birth weight z-scores between −1.645 to 1.645, small for gestational age (SGA) as birth weight z-scores <−1.645, and large for gestational age (LGA) as birth weight z-scores >1.645.

Systolic and diastolic blood pressure percentiles according to sex, age, and height percentile were determined via an online age-based pediatric blood pressure calculator [27]. The pubertal stage was graded according to Marshall and Tanner [28, 29]. Puberty onset was defined as genitalia Tanner stage 2 in boys with a testicular volume >3 mL and breast bud appearance in girls. Full puberty was indicated when pubertal signs matched Tanner stage 5.

#### Body composition assessment

Body composition was assessed by BIA (Tanita Body-Composition Analyzer, Tanita MC-780 MA, and GMON Professional Software) [30]. The BIA covered both the entire body as well as segmental analyses (trunk, upper and lower limbs) for fat and muscle. Measurements took place during routine clinic visits, preferably in the morning (8:00 AM to 1:00 PM), with subjects in a fasting, non-exerted state. The process involved standing barefoot on the analyzer and gripping the handles, and it took roughly 1 minute per subject. The BIA report contained data on whole body and segmental analysis of fat and muscle: fat percentage (FATP), fat mass, and muscle mass. Calculated variables included appendicular skeletal muscle mass (ASMM, the sum of limb muscle mass) and muscle-to-fat ratio (MFR = ASMM/fat mass). Z-scores for ASMM and MFR were determined by BIA pediatric reference curves [31].

### Statistical analysis

Data were analyzed by IBM SPSS software (IBM SPSS Statistics for Windows, Version 29; IBM Corp., Armonk, NY). Continuous data were presented as mean ± standard deviation (normal distribution) or median [interquartile range] (skewed distribution), and as number and percentage for categorical variables. Differences in continuous data between groups were examined by independent-sample t-tests (normally distributed data) or Mann–Whitney U-tests (skewed data). Fisher’s exact and Chi-squared tests were used to examine the differences in categorical data, as appropriate. Generalized estimating equation (GEE) linear models were used to explore the contribution of clinical factors (sex, study group, age, height z-score, delta height z-score, cumulative rhGH dose, and IGF1 z-score) to the body composition components (FATP, MFR z-score, and ASMM z-score). The statistical findings are based on all cases with valid data for all the variables in the model. The GEE model was chosen in order to produce regression estimates when analyzing repeated measures. A *p*-value of ≤0.05 was considered significant.

## Results

**Fig. 1:**
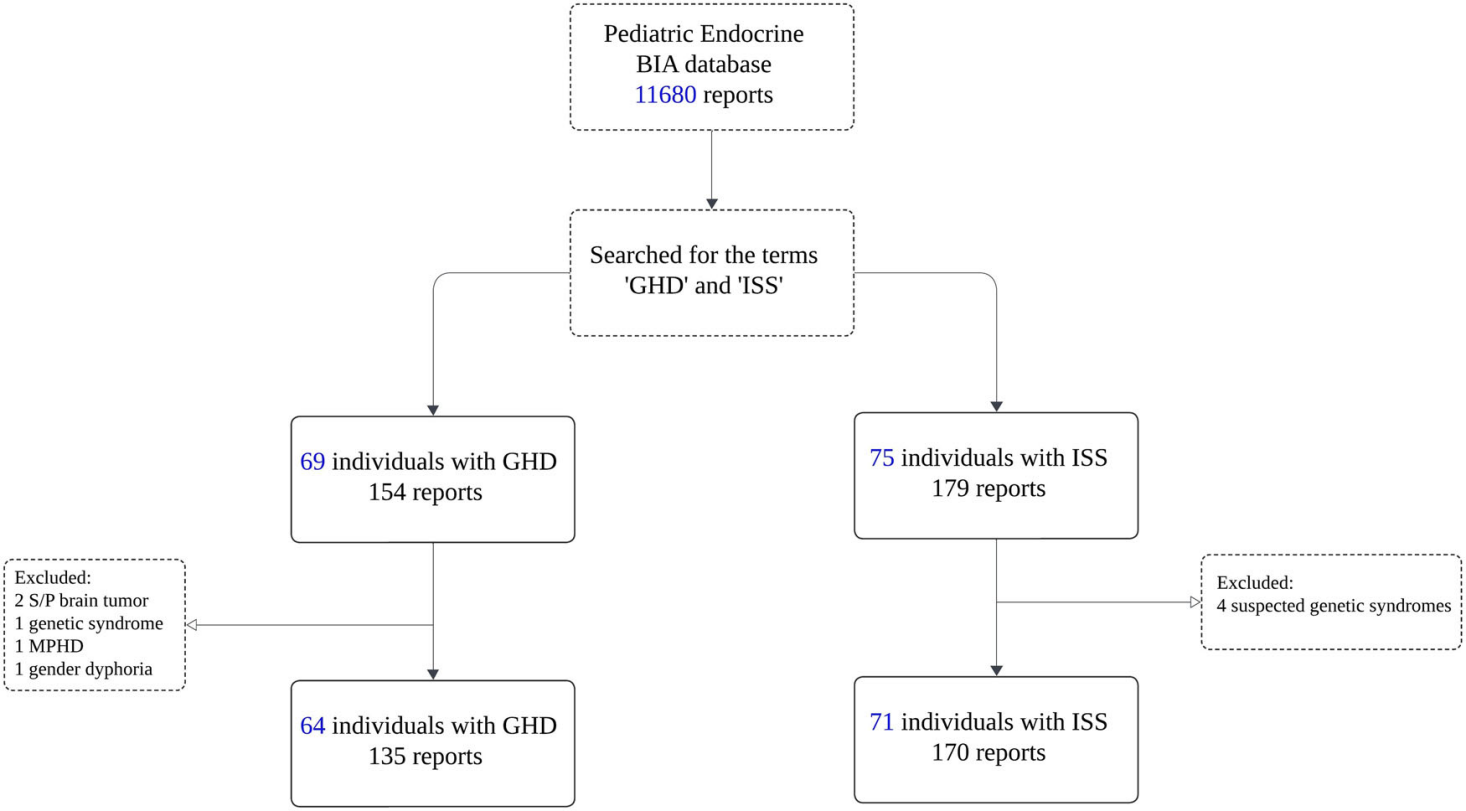
The study flowchart. Growth hormone deficiency; ISS, idiopathic short stature; MPHD, multiple pituitary hormone deficiency

The analyses included 305 BIA reports of 135 pediatric patients (89 boys, 65.9%), of whom 64 were diagnosed with GHD and 71 with ISS (Fig. 1). All patients in the GHD group underwent brain MRI as part of the routine evaluation of GHD, and the findings were abnormal in 11 (17.2%): ectopic pituitary and/or abnormal stalk (*n* = 4), small pituitary gland (*n* = 3), pituitary microadenoma (*n* = 1), Rathke cleft cyst (*n* = 1), and Chiari 1 (*n* = 2). Twenty-eight girls (60.8%) underwent karyotype analysis, all with normal findings.

Sociodemographic and medical background characteristics of patients stratified by study group (GHD or ISS) are presented in Table 1. The mean age of the entire cohort was 10.7 ± 3.0 years (range 5–17.2 years), of whom 74 (54.8%) were prepubertal and 61 (45.2%) were pubertal (Tanner stages 2–5). Their collective SEP was above average: the median SEP cluster was 8 (range 1 to 10), with a median SEP index of 1.378 (range −1.719 to 2.765). The patients’ median gestational age was 40 (range 25 to 42), with 20 (14.8%) having been born preterm. Most of the cohort were born AGA (*n* = 104. 77%), with a normal mean birth weight that had a wide standard deviation [−0.91 ± 1.30]. Eight children (5.9%) had celiac disease with negative celiac serology under a gluten-free diet, and 5 children (3.7%) were diagnosed with ADHD and treated by stimulants. There were no significant group differences in sociodemographic characteristics or medical background. Family history of cardiovascular risk factors (obesity, hypertension, type 2 diabetes, and dyslipidemia) also did not differ between the groups. Of note, while no significant group difference was found in the systolic blood pressure percentile, the diastolic blood pressure percentile was significantly higher in the GHD group (*p* = 0.050).

**Table 1:** Sociodemographic and clinical characteristics of patients with growth hormone deficiency (GHD) or idiopathic short stature (ISS)

Parameter	GHD, *n* = *64*	ISS, *n* = 71	*p*-value
Males, *n* (%)	42 (65.6)	47 (66.2)	0.944
Mid-parental height, z-score	−0.28 [−1.04, 0.05]	−0.60 [−1.34, −0.05]	**0.048**
Socioeconomic position (SEP)			
SEP cluster	8 [7,9]	8 [7,9]	0.845
SEP index	1.346 [0.769, 1.876]	1.396 [0.860, 1.953]	0.874
Birth characteristics			
Gestational age (wks)	40 [38,40]	39 [38,40]	0.456
Prematurity, *n* (%)	11 (17.2)	9 (12.7)	0.479
Birth weight z-scores	0.69 ± 1.15	−1.11 ± 1.40	0.331
Birth weight categories			
SGA, *n* (%)	13 (20.3)	17 (23.9)	0.308
AGA, *n* (%)	51 (79.7)	53 (74.6)	
LGA, *n* (%)	0 (0)	1 (1.4)	
Age (yrs)	10.8 ± 2.9	10.7 ± 3.1	0.528
Pubertal stage			
Prepubertal (Tanner 1)	35 (54.7)	39 (54.9)	0.817
In puberty (Tanner 2–5)	29 (45.3)	32 (45.1)	
Blood pressure (BP) percentiles			
Systolic BP	68.0 [54.8, 84.0]	69.0 [44.0, 81.0]	0.314
Diastolic BP	67.5 [52.0, 76.5]	57.0 [41.0, 73.0]	**0.050**
Comorbid conditions			
Attention deficit disorder	5 (7.8)	3 (4.2)	0.467
Celiac disease	1 (1.5)	4 (5.6)	0.367

Data are expressed as number and (percent), median [interquartile range], or mean ± standard deviation. Chi-squared tests were performed to compare categorical variables between groups, and the Mann-Whitney test was performed to compare linear variables with skewed distribution. *Prematurity* gestational age ≤37 weeks; *SGA* small for gestational age, *AGA* appropriate for gestational age, *LGA* large for gestational age. A *p-*value ≤ 0.05 was considered significant.

*P* values that statistically significant are shown in bold.

The genetic height potential, as expressed by the MPH z-score, was taller in the GHD group compared to the ISS group (*p* < 0.001). At their first BIA, the subjects in the GHD group were significantly taller than those in the ISS group (*p* < 0.001). There was a significant association between the height z-score and the MPH z-score (*r* = 0.319, *p* < 0.001) of all the patients in the entire cohort. The achievement of the genetic potential, as expressed by delta height z-scores, did not differ between the groups.

**Fig. 2:**
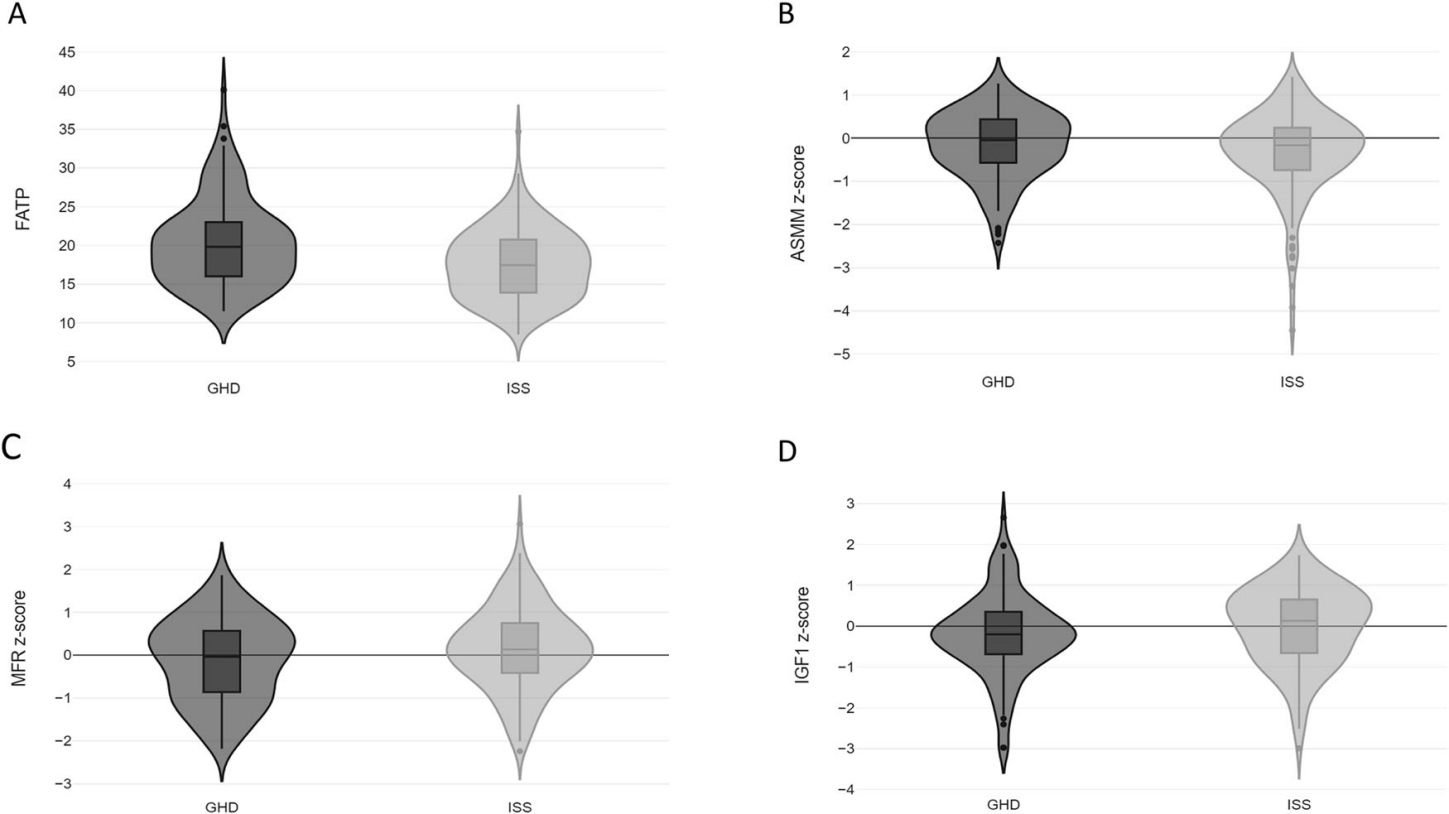
Violin plots for the distribution of body composition components and insulin-like growth factor 1 in pediatric patients with growth hormone deficiency and those with idiopathic short stature at first BIA. **A** Fat percentage (FATP); **B** appendicular skeletal muscle mass (ASMM) z-score; **C** Muscle-to-fat (MFR) z-score; **D** IGF1 z-score

At first BIA, the vast majority of the cohort (*n* = 116; 85.9%) was in the normal weight category. However, patients in the GHD group had significantly higher BMI z-scores compared to those in the ISS group (*p* < 0.001). Comparisons between body composition components of the two study groups revealed higher fat percentage, higher truncal fat percentage, and lower MFR z-score in the GHD group (*p* = 0.010, *p* = 0.027, and *p* = 0.050, respectively). The muscle component (represented by the ASMM z-score) of the entire cohort was −0.40 ± 0.99 and it did not differ between the groups. Figure 2A–C displays the distribution of body composition components (FATP, MFR z-scores, and ASMM z-scores) in the GHD and ISS groups at the first BIA assessment.

The clinical characteristics, anthropometric measurements, and body composition components of the patients at first BIA evaluation, stratified according to diagnosis and according to rhGH treatment at first BIA, are presented in Table 2. At first BIA, 90 (66.7%) patients had already been treated by rhGH. In non-treated patients, the FATP and truncal FATP were significantly higher in the GHD group compared to ISS group (*p* = 0.002 and *p* = 0.008, respectively), but not significant in the rhGH-treated patients. The rhGH-treated patients in both the GHD and ISS groups had lower truncal FATP (*p* < 0.001, *p* = 0.004, respectively). Finally, the ASMM z-score was higher in the rhGH-treated patients in both the GHD and ISS groups (*p* < 0.001 for both).

**Table 2:** Characteristics of patients with growth hormone deficiency (GHD) or idiopathic short stature (ISS) at first bioimpedance analysis assessment (BIA), classified as treated versus not treated with rhGH at first BIA

	Not treated with rhGH			Treated with rhGH				
Parameter	GHD	ISS	*p*_1_-value^*^	GHD	ISS	*p*_2_-value^*^	*p*_3_-value^*^	*p*_4_-value^*^
Patients, *n*	22	23		42	48			
Males, *n* (%)	13 (59.1)	19 (82.6)	0.107	29 (69)	28 (58.3)	0.381	0.580	0.061
Age (yrs)	10.6 ± 2.8	9.2 ± 3.2	0.116	10.8 ± 3.0	11.5 ± 2.8	0.32	0.784	**0.003**
Anthropometric measurements								
Height, z-score	−1.19 [−2.04, −1.42]	−2.20 [−2.38, −19.3]	**0.003**	−0.59 [−1.29, −0.15]	−1.36 [−1.82, −0.88]	**<0.001**	**<0.001**	**<0.001**
Delta height, z-score	−1.43 [−1.95, −0.76]	−1.22 [−1.71, −0.66]	0.447	−0.25 [−0.92, 0.40]	−0.67 [−1.58, −0.16]	**0.002**	**<0.001**	**0.027**
Body mass index, z-score	0.20 [−0.45, 1.31]	−0.70 [−1.47, 0.24]	**0.008**	−0.38 [−1.00, 0.49]	−0.49 [−1.18, 0.14]	0.363	**0.031**	0.69
Body Composition								
Fat percentage	23.75 [21.38, 29.23]	20.50 [17.30, 21.80]	**0.002**	18.25 [16.15, 22.28]	18.00 [14.60, 20.58]	0.241	**<0.001**	0.058
Truncal fat percentage	18.9 [16.10, 24.60]	15.6 [12.20, 18.00]	**0.008**	12.6 [11.20, 16.20]	12.7 [10.30, 14.70]	0.282	**<0.001**	**0.004**
Appendicular skeletal muscle mass, z-score	−1.00 [−1.59, −0.41]	−1.1 [−2.50, −0.72]	0.180	−0.02 [−0.47, 0.45]	0.29 [−0.39, 0.52]	0.442	**<0.001**	**<0.001**
Muscle-to-fat ratio, z-score	−1.06 [−1.52, −0.47]	−1.03 [−1.35, −0.30]	0.699	0.00 [−0.35, 0.53]	0.41 [−0.19, 0.84]	**0.040**	**<0.001**	**<0.001**
IGF1 z-score	−0.88 [−1.40, −0.40]	−0.65 [−0.99, −0.20]	0.308	0.00 [−0.23, 0.63]	0.27 [−0.38, 0.65]	0.472	**<0.001**	**<0.001**

Data are expressed as number and (percent) or median [interquartile range]. Chi-squared tests compared categorical variables between groups, and the Mann-Whitney test compared linear variables with skewed distribution. A *p* value of ≤0.05 was considered significant.

*p1-value: comparison between GHD and ISS patients not treated with rhGH, *p_2_-value: comparison between GHD and ISS patients treated with rhGH, *p_3_-value: comparison between rhGH treated vs not treated GHD patients, *p_4_-value: comparison between rhGH treated vs not treated ISS patients.

*P* values that statistically significant are shown in bold.

In 239 (78.4%) of BIA assessments, patients had been treated by rhGH, for a mean treatment duration of 3.7 years, without significant difference between the groups (*p* = 0.903). Forty-three (67.2%) patients with GHD had received a median dose of 31 mcg/kg/dy [IQR 27.0, 37.5], while 47 (66.2%) patients with ISS had received 40 mcg/kg/dy [IQR 33.0, 43.0], *p* < 0.001. The mean IGF1 z-score at BIA assessment for the entire cohort was −0.10 ± 0.97, and the distribution of IGF1 z-scores of the GHD and ISS groups at their first BIA assessment is presented in Fig. 2D. The IGF1 z-score was significantly higher in both the GHD and ISS patients who were treated by rhGH (*p* < 0.001 for both groups). Only one patient diagnosed with GHD had an elevated IGF1 z-score of 2.67 at 7 months of treatment, which subsequently normalized during follow-up after rhGH dose adjustment.

GEE linear models were applied for identification of the contributing factors to the body composition components (Table 3). A higher FATP was associated with female sex (*p* < 0.001), GHD diagnosis (*p* < 0.001), lower height z-score (*p* = 0.036), and greater delta height z-score (*p* = 0.041). SEP index, age, cumulative rhGH dose, and IGF1 z-scores were not significant in the GEE model for FATP. A higher MFR z-score was associated with female sex (*p* = 0.049), an ISS diagnosis (*p* = 0.005), a higher height z-score (*p* = 0.004), and a smaller delta height z-score (*p* = 0.048). SEP index, age, cumulative rhGH dose, and IGF1 z-scores were not significant in the GEE model for the MFR z-scores. A higher ASMM z-score was associated with female sex (*p* < 0.001), older age (*p* < 0.001), higher height z-score (*p* = 0.003), and higher IGF1 z-score (*p* = 0.021). SEP index, study group, delta height z-score, and cumulative dose rhGH were not significant in the GEE model for the ASMM z-score. Females demonstrated a greater increase in IGF1 z-score over time after rhGH cumulative dose adjustment (β = 0.44, SE = 0.16, *p* = 0.006).

**Table 3:** Generalized estimating equations (GEE) linear models applied to evaluate contributory factors for body composition parameters: fat percentage (FATP), muscle-to-fat ratio (MFR) z-score and appendicular skeletal muscle mass (ASMM) z-score

					95% Confidence interval	
	Constants	β	SE	*p* value	Lower	Upper
FATP	Sex (F/M)	4.179	0.697	**<0.001**	2.813	5.545
	Group (GHD/ISS)	2.918	0.818	**<0.001**	1.314	4.521
	Age	−0.010	0.133	0.943	−0.269	0.250
	Height z-score	−1.252	0.597	**0.036**	−2.422	−0.081
	Delta height z-score	1.210	0.592	**0.041**	0.050	2.371
	IGF1 z-score	−0.750	0.550	0.173	−1.828	0.328
MFR z-score	Sex (F/M)	0.275	0.1396	**0.049**	0.020	0.548
	Group (GHD/ISS)	−0.444	0.157	**0.005**	−0.752	−0.136
	Age	0.050	0.029	0.086	−0.007	0.106
	Height z-score	0.304	0.110	**0.004**	0.101	0.533
	Delta height z-score	−0.213	0.107	**0.048**	−0.002	3.924
	IGF1 z-score	0.070	0.109	0.369	−0.083	0.224
ASMM z-score	Sex (F/M)	0.446	0.117	**<0.001**	0.216	0.675
	Group (GHD/ISS)	−0.025	0.123	0.836	−0.265	0.215
	Age	0.084	0.025	**<0.001**	0.035	0.134
	Height z-score	0.235	0.078	**0.003**	0.081	0.388
	Delta height z-score	−0.100	0.074	0.174	−0.244	0.044
	IGF1 z-score	0.170	0.074	**0.021**	0.025	0.314

Variables entered into the models: sex, socioeconomic position (SEP) index, group, age, height z-score, delta height z-score, cumulative rhGH dose, and IGF1 z-score. SEP index and cumulative rhGH dose results were not significant in the models. A *p* value of ≤0.05 was considered significant.

*P* values that statistically significant are shown in bold.

## Discussion

To the best of our knowledge, our study is the first to examine the relationship between IGF1 levels and body composition components of fat and muscle in pediatric patients with ISS under treatment with rhGH. We found that individuals diagnosed with GHD exhibited increased adiposity characterized by a higher fat percentage, a higher truncal fat percentage, and a compromised muscle-to-fat ratio z-score compared to those with ISS. Of note, the muscle component (ASMM z-scores) did not differ between the GHD and ISS groups. Given that the IGF1 levels in patients play a significant role in determining their muscle mass, we propose that the rhGH treatment that is administered with the aim of restoring normal IGF1 levels may potentially mitigate the detrimental metabolic impact associated with increased adiposity.

There is no argument that height has a strong heritable trait [32], as had been supported by our results demonstrating the link between patients’ height z-scores and their mid-parental height z-scores, even for patients under rhGH treatment. Notably, our ISS patients were of shorter stature compared to those with GHD, a finding that was consistent with a shorter familial height. As a result, the comparison of delta height z-score, which indicates the achievement of genetic height during treatment, showed no difference between the two groups. These observations are in line with the objectives of rhGH treatment being different for each group: the primary goal of rhGH treatment for GHD is to attain the individual’s genetic height potential, while the primary focus of the treatment for ISS is upon achieving a height within or above the third percentile. Of note, patients with ISS were treated with a higher dose of rhGH compared to patients with GHD and attained similar IGF1 levels. This may imply a variable degree of GH resistance in patients with ISS, which may limit the effect of rhGH therapy [33].

Genetic predisposition has also been identified in body composition parameters [34], and genetic influence may contribute to the hereditary nature of metabolic disorders [35]. Both of the current study groups exhibited a similar low prevalence of familial obesity and metabolic syndrome components, suggesting similarity in metabolic risk. Most of our patients with GHD had already been treated by rhGH, and they were characterized by a higher fat percentage, a higher truncal fat percentage, and higher diastolic blood pressure percentiles than our patients in the ISS group. The statistical model we applied found that the diagnosis of GHD was related to increased adiposity, after adjusting for cumulative rhGH dose. These findings point towards a metabolic role of endogenous GH and raise several questions regarding the extent of reduction in adiposity, the time required for such reduction, and whether normal adiposity levels are attainable.

Increased adiposity, especially in visceral locations, has been reported in both adult and pediatric patients with GHD prior to treatment initiation [36–38]. Studies in adults found a marked reduction in total body fat content under treatment with rhGH [38–41] and, more specifically, in visceral adiposity [42–44], attributed to the lipolytic effect of GH. Poor compliance with treatment, marked by frequent and extended breaks, reportedly led to deteriorating metabolic parameters [45]. A reduction in adiposity had been noted mostly in the first months of treatment, with contradicting reports on the sustainability of achievement in pediatric patients [46, 47]. Examining the body composition of adults who had received rhGH treatment during childhood for isolated GHD could contribute valuable insight into issue.

The primary concern with increased adiposity is its association with cardiovascular disease risk. This link has primarily been shown in adults, with a limited number of studies indicating a similar association in pediatric populations [15]. In the current study, we observed a higher diastolic blood pressure percentile in patients with GHD but none in the systolic blood pressure percentile. This could herald an increased metabolic risk which could stem from an unfavorable body composition. The MFR z-score, which accounts for the muscle-to-fat ratio adjusted for sex and age, was lower in our GHD group. We had earlier demonstrated the strong predictive value of the MFR z-score in the detection of cardiometabolic risk in pediatric patients with obesity, type 1 diabetes, and non-classic congenital adrenal hyperplasia [14, 16, 48]. We now consider that employing therapeutic approaches that incorporate rhGH and that aim to address both fat and muscle components of body composition may decrease potential metabolic risks in a pediatric GHD population.

Sexual dimorphism could influence the way a medical condition is presented, progresses, and responds to treatment. There are variations in body composition between the sexes already at birth, with both males and females exhibiting comparable fat mass while males typically exhibit greater length and a higher proportion of lean mass [49]. As individuals progress into adulthood, boys undergo an increase in muscle mass with a concurrent decrease in fat, whereas females exhibit a lower propensity for muscle development and are more likely to accumulate peripheral fat [50]. In line with expectations, our study identified female sex as a contributing factor for increased adiposity. Surprisingly, however, female sex was identified as a contributor to a favorable sex- and age-adjusted muscle mass (ASMM z-score), as well as to a better muscle-to-fat ratio z-score.

The acquisition of skeletal muscle is affected by a complex interplay between genetic, hormonal, and environmental factors [51]. Of note, all of our patients are provided with nutritional counseling and recommended to routinely participate in physical activity according to the WHO guidelines for the pediatric population [52]. Since lifestyle habit questionnaires were not part of this study, we cannot comment upon the possibility that behavioral differences between the sexes account for more advantageous sex- and age-adjusted muscle mass among the females. Differences in the response to rhGH therapy had been observed between adult men and women with GHD [53] and sex hormone-dependent anabolic effects of GH could partially explain some of our findings in the pediatric population. The exact biological mechanisms by which sex steroid patterns could be a contributing factor, however, remain obscure. One report in the pediatric population suggested that sex hormones may lead to distinct GH responsiveness by influencing the production of IGF1 [54]. This is supported by our finding of greater responsiveness to rhGH therapy in females as manifested by a greater increase in IGF1 z-scores over time.

We demonstrated that sex- and age-adjusted IGF1 levels related to muscle mass but not to adiposity. It has been previously reported that IGF1 plays a critical role in the regulation of protein metabolism by enhancing the transport of amino acids into muscle cells, thus supporting a continuous supply of components for protein synthesis [3, 6]. Furthermore, it inhibits the degradation of pre-existing proteins within muscle tissue. By doing so, IGF1 makes a substantial contribution to the preservation and expansion of skeletal muscle, influencing both muscle size and strength [55, 56]. IGF1 has been reported to also possess metabolic and health-promoting capabilities, such as atheroprotective, neuroprotective, and insulin-like effects [57]. However, the myotrophic effect of IGF1 may be attenuated by insulin resistance in the case of increased adiposity [4]. Given that the cumulative dose of rhGH in our patients did not have a direct impact on body composition parameters, it can be inferred that the individual responsiveness to rhGH, mediated by endogenous IGF1 secretion, is the more likely factor influencing muscle mass.

The study has several limitations, such as its cross-sectional design, which precludes our ability to establish a causal relationship between exposure and outcomes. Additionally, it may not be representative of the pediatric GHD and ISS populations, since it includes only youth who visit our facilities. Lastly, the reliance on self-reported family histories of cardiovascular risk factors may introduce potential bias due to accuracy and recall issues. The primary strength of this study derives from the standardized and comprehensive assessment by trained medical experts, which supports the reliability of measurements, including those of blood pressure, anthropometrics, pubertal status, and body composition assessment. Another strength lies in the uniformity of medical care provided by the same multi-professional team in a single hospital-based tertiary center and the comprehensiveness of patient follow-up, including medical nutrition therapy and promotion of a healthy lifestyle.

## Conclusions

The findings of our study demonstrate an inclination towards greater adiposity in young individuals with GHD compared to those with ISS. This is manifested by higher fat percentages, truncal fat percentages, and an altered muscle-to-fat ratio z-score, while the muscle component remained unaffected. IGF1 levels were not found to be significantly associated with body fat percentage, suggesting a mechanism not linked to IGF1 in the determination of body fat. Given the pivotal role of IGF1 in muscle mass regulation, we consider that rhGH treatment targeting normal IGF1 levels may offer a potential approach to mitigate the increased adiposity in patients with GHD by means of promoting muscle growth.

## References

[1] A. Grimberg, D.B. Allen, Growth hormone treatment for growth hormone deficiency and idiopathic short stature: new guidelines shaped by the presence and absence of evidence. Curr. Opin. Pediatr. **29**, 466–471 (2017). 10.1097/MOP.0000000000000505 28525404 10.1097/MOP.0000000000000505 PMC5565215

[2] P. Wang, B. Ji, Q. Shao, M. Zhang, B. Ban: Association between insulin-like growth factor-1 and uric acid in Chinese children and adolescents with idiopathic short stature: a cross-sectional study. Biomed Res. Int. **2018**, (2018). 10.1155/2018/4259098 10.1155/2018/4259098 PMC5896336 29789791

[3] C. Duan, H. Ren, S. Gao, Insulin-like growth factors (IGFs), IGF receptors, and IGF-binding proteins: Roles in skeletal muscle growth and differentiation. Gen. Comp. Endocrinol. **167**, 344–351 (2010). 10.1016/J.YGCEN.2010.04.009 20403355 10.1016/j.ygcen.2010.04.009

[4] A. Brener et al. Insulin-like growth factor-1 status is associated with insulin resistance in young patients with spinal muscular atrophy. Neuromuscul. Disord. **30**, 888–896 (2020). 10.1016/j.nmd.2020.09.025 33071067 10.1016/j.nmd.2020.09.025

[5] C.P. Hawkes, A. Grimberg, Insulin-like growth Factor-I is a marker for the nutritional state. Pediatr. Endocrinol. Rev. **13**, 499 (2015) 26841638 PMC5576178

[6] T.V. Bersin et al. Nutritional status affects Igf1 regulation of skeletal muscle myogenesis, myostatin, and myofibrillar protein degradation pathways in gopher rockfish (Sebastes carnatus). Mol. Cell. Endocrinol. **573**, 111951 (2023). 10.1016/J.MCE.2023.111951 37169322 10.1016/j.mce.2023.111951

[7] A. Pawlikowska-Haddal, P. Cohen, D.M. Cook, How useful are serum IGF-I measurements for managing GH replacement therapy in adults and children? Pituitary **15**, 126–134 (2012). 10.1007/S11102-011-0343-Y/TABLES/5 21909971 10.1007/s11102-011-0343-y

[8] A. Grimberg et al. Guidelines for growth hormone and insulin-like growth Factor-I treatment in children and adolescents: growth hormone deficiency, idiopathic short stature, and primary insulin-like growth Factor-I deficiency. Horm. Res. Paediatr. **86**, 361–397 (2017). 10.1159/000452150 10.1159/000452150 27884013

[9] A. Brener et al. Beyond Body Mass Index - Body composition assessment by bioimpedance in routine endocrine practice. Endocr. Pract. **27**, 419–425 (2021). 10.1016/j.eprac.2020.10.013 33934752 10.1016/j.eprac.2020.10.013

[10] S.D. De Ferranti et al. Prevalence of the metabolic syndrome in American adolescents: findings from the Third National Health and Nutrition Examination Survey. Circulation **110**, 2494–2497 (2004). 10.1161/01.CIR.0000145117.40114.C7 15477412 10.1161/01.CIR.0000145117.40114.C7

[11] G.E. Duncan, S.M. Li, X.H. Zhou, Prevalence and trends of a metabolic syndrome phenotype among U.S. adolescents, 1999-2000. Diabetes Care **27**, 2438–2443 (2004). 10.2337/DIACARE.27.10.2438 15451913 10.2337/diacare.27.10.2438

[12] J. Steinberger et al. Progress and challenges in metabolic syndrome in children and adolescents. Circulation **119**, 628–647 (2009). 10.1161/CIRCULATIONAHA.108.191394 19139390 10.1161/CIRCULATIONAHA.108.191394

[13] A. Brener et al. The endocrine manifestations of spinal muscular atrophy, a real-life observational study. Neuromuscul. Disord. **30**, 270–276 (2020). 10.1016/j.nmd.2020.02.011 32273202 10.1016/j.nmd.2020.02.011

[14] N. Salton et al. Muscle-to-fat ratio for predicting metabolic syndrome components in children with overweight and obesity. Child. Obes. **18**, 132–142 (2022). 10.1089/CHI.2021.0157 34550798 10.1089/chi.2021.0157

[15] A. Yerushalmy-Feler et al. Body composition in pediatric celiac disease and metabolic syndrome component risk-an observational study. Pediatr. Res. **94**, 618–625 (2023). 10.1038/S41390-023-02496-3 36707663 10.1038/s41390-023-02496-3

[16] A. Ben Simon et al. Body composition in children and adolescents with non-classic congenital adrenal hyperplasia and the risk for components of metabolic syndrome: An observational study. Front. Endocrinol. **13**, 1022752 (2022). 10.3389/FENDO.2022.1022752/BIBTEX 10.3389/fendo.2022.1022752 PMC9639453 36353234

[17] Israel Central Bureau of Statistics. Characterization and Classification of Geographical Units by the Socio-Economic Level of the Population, 2015. (2020). https://www.cbs.gov.il/he/publications/DocLib/2020/1765_socio_economic_2015/e_print.pdf.

[18] A. Grimberg, C.P. Hawkes, Growth Hormone Treatment for Non-GHD Disorders: Excitement Tempered by Biology. J. Clin. Endocrinol. Metab. **109**, e442–e454 (2024). 10.1210/clinem/dgad417 37450564 10.1210/clinem/dgad417 PMC10795916

[19] M. Yackobovitch-Gavan, L. Lazar, R. Diamant, M. Phillip, T. Oron, Diagnosis of growth hormone deficiency in children: the efficacy of glucagon versus clonidine stimulation test. Horm. Res. Paediatr. **93**, 470–476 (2021). 10.1159/000513393 10.1159/000513393 33567442

[20] M. Bidlingmaier et al. Reference intervals for insulin-like growth factor-1 (igf-i) from birth to senescence: results from a multicenter study using a new automated chemiluminescence IGF-I immunoassay conforming to recent international recommendations. J. Clin. Endocrinol. Metab. **99**, 1712–1721 (2014). 10.1210/JC.2013-3059 24606072 10.1210/jc.2013-3059

[21] P. Chanson et al. Reference values for IGF-I Serum concentrations: comparison of six immunoassays. J. Clin. Endocrinol. Metab. **101**, 3450–3458 (2016). 10.1210/JC.2016-1257 27167056 10.1210/jc.2016-1257 PMC5054194

[22] R.J. Kuczmarski et al.: CDC growth charts: United States. Adv. Data 1-27 (2000). https://stacks.cdc.gov/view/cdc/11267 11183293

[23] J.M. Tanner, H. Goldstein, R.H. Whitehouse, Standards for children’s height at ages 2-9 years allowing for height of parents. Arch. Dis. Child. **45**, 755–762 (1970). 10.1136/ADC.45.244.755 5491878 10.1136/adc.45.244.755 PMC1647404

[24] S.E. Barlow, W.H. Dietz, Obesity evaluation and treatment: Expert Committee recommendations. The Maternal and Child Health Bureau, Health Resources and Services Administration and the Department of Health and Human Services. Pediatrics **102**, e29 (1998). 10.1542/PEDS.102.3.E29 9724677 10.1542/peds.102.3.e29

[25] T.J. Cole, K.M. Flegal, D. Nicholls, A.A. Jackson, Body mass index cut offs to define thinness in children and adolescents: international survey. BMJ **335**, 194–197 (2007). 10.1136/BMJ.39238.399444.55 17591624 10.1136/bmj.39238.399444.55 PMC1934447

[26] J.H. Chou, S. Roumiantsev, R. Singh: PediTools electronic growth chart calculators: applications in clinical care, research, and quality improvement. J. Med. Internet Res. **22**, (2020). 10.2196/16204 10.2196/16204 PMC7058170 32012066

[27] Shypailo R.J.: Age-Based Pediatric Blood Pressure Reference Charts. from the Baylor College of Medicine, Children’s Nutrition Research Q21 Center, Body Composition Laboratory. (2018). http://www.bcm.edu/bodycomplab/BPappZjs/BPvAgeAPPz.html

[28] W.A. Marshall, J.M. Tanner, Variations in the pattern of pubertal changes in boys. Arch. Dis. Child. **45**, 13–23 (1970). 10.1136/ADC.45.239.13 5440182 10.1136/adc.45.239.13 PMC2020414

[29] W.A. Marshall, J.M. Tanner, Variations in pattern of pubertal changes in girls. Arch. Dis. Child. **44**, 291–303 (1969). 10.1136/ADC.44.235.291 5785179 10.1136/adc.44.235.291 PMC2020314

[30] C.E. Orsso, M.C. Gonzalez, M.J. Maisch, A.M. Haqq, C.M. Prado, Using bioelectrical impedance analysis in children and adolescents: Pressing issues. Eur. J. Clin. Nutr. **76**, 659–665 (2022). 10.1038/S41430-021-01018-W 34620999 10.1038/s41430-021-01018-w

[31] H.D. McCarthy, D. Samani-Radia, S.A. Jebb, A.M. Prentice, Skeletal muscle mass reference curves for children and adolescents. Pediatr. Obes. **9**, 249–259 (2014). 10.1111/J.2047-6310.2013.00168.X 23776133 10.1111/j.2047-6310.2013.00168.x

[32] L. Lello et al. Accurate genomic prediction of human height. Genetics **210**, 477–497 (2018). 10.1534/GENETICS.118.301267 30150289 10.1534/genetics.118.301267 PMC6216598

[33] M.O. Savage, H.L. Storr, G.H. Resistance, Is a component of idiopathic short stature: implications for rhGH therapy. Front Endocrinol. **12**, 781044 (2021). 10.3389/FENDO.2021.781044/BIBTEX 10.3389/fendo.2021.781044 PMC8702638 34956092

[34] A. Brener et al. The heritability of body composition. BMC Pediatr. **21**, 1–8 (2021). 10.1186/S12887-021-02695-Z/FIGURES/1 33964919 10.1186/s12887-021-02695-z PMC8105919

[35] G.A. Bray, R.M. Krauss, F.M. Sacks, L. Qi, Lessons learned from the POUNDS lost study: genetic, metabolic, and behavioral factors affecting changes in body weight, body composition, and cardiometabolic risk. Curr. Obes. Rep. **8**, 262–283 (2019). 10.1007/S13679-019-00353-1/TABLES/3 31214942 10.1007/s13679-019-00353-1

[36] S.A. Beshyah et al. Abnormal body composition and reduced bone mass in growth hormone deficient hypopituitary adults. Clin. Endocrinol. **42**, 179–189 (1995). 10.1111/J.1365-2265.1995.TB01860.X 10.1111/j.1365-2265.1995.tb01860.x 7704962

[37] G. Johannsson et al. Growth hormone treatment of abdominally obese men reduces abdominal fat mass, improves glucose and lipoprotein metabolism, and reduces diastolic blood pressure. J. Clin. Endocrinol. Metab. **82**, 727–734 (1997). 10.1210/JCEM.82.3.3809 9062473 10.1210/jcem.82.3.3809

[38] D. Maiter et al. Baseline characteristics and response to GH replacement of hypopituitary patients previously irradiated for pituitary adenoma or craniopharyngioma: data from the Pfizer International Metabolic Database. Eur. J. Endocrinol. **155**, 253–260 (2006). 10.1530/EJE.1.02209 16868138 10.1530/eje.1.02209

[39] F. Salomon, R.C. Cuneo, R. Hesp, P.H. Sönksen, The effects of treatment with recombinant human growth hormone on body composition and metabolism in adults with growth hormone deficiency. N. Engl. J. Med. **21**, 1797–1803 (2010). 10.1056/NEJM198912283212605 10.1056/NEJM198912283212605 2687691

[40] A.F. Attanasio et al. Human growth hormone replacement in adult hypopituitary patients: long-term effects on body composition and lipid status—3-year results from the HypoCCS database. J. Clin. Endocrinol. Metab. **87**, 1600–1606 (2002). 10.1210/JCEM.87.4.8429 11932289 10.1210/jcem.87.4.8429

[41] N. Moøller, J.O.L. Joørgensen, Effects of growth hormone on glucose, lipid, and protein metabolism in human subjects. Endocr. Rev. **30**, 152–177 (2009). 10.1210/ER.2008-0027 19240267 10.1210/er.2008-0027

[42] A. Chrisoulidou et al. Effects of 7 years of growth hormone replacement therapy in hypopituitary adults. J. Clin. Endocrinol. Metab. **85**, 3762–3769 (2000). 10.1210/JCEM.85.10.6910 11061536 10.1210/jcem.85.10.6910

[43] C. Franco et al. The reduction in visceral fat mass in response to growth hormone is more marked in men than in oestrogen-deficient women. Growth Horm. IGF Res. **19**, 112–120 (2009). 10.1016/J.GHIR.2008.07.001 18752977 10.1016/j.ghir.2008.07.001

[44] A.R. Hoffman et al. Growth hormone (GH) replacement therapy in adult-onset gh deficiency: effects on body composition in men and women in a double-blind, randomized, placebo-controlled trial. J. Clin. Endocrinol. Metab. **89**, 2048–2056 (2004). 10.1210/JC.2003-030346 15126520 10.1210/jc.2003-030346

[45] Y.J. Lee et al. Metabolic impacts of discontinuation and resumption of recombinant human growth hormone treatment during the transition period in patients with childhood-onset growth hormone deficiency. Endocrinol. Metab. **37**, 359–368 (2022). 10.3803/ENM.2021.1384 10.3803/EnM.2021.1384 PMC9081298 35504604

[46] I.M. Van Der Sluis et al. Long-term effects of growth hormone therapy on bone mineral density, body composition, and serum lipid levels in growth hormone deficient children: a 6-year follow-up study. Horm. Res. **58**, 207–214 (2002). 10.1159/000066262 12401939 10.1159/000066262

[47] J.N. Roemmich, M.G. Huerta, S.M. Sundaresan, A.D. Rogol, Alterations in body composition and fat distribution in growth hormone—Deficient prepubertal children during growth hormone therapy. Metabolism **50**, 537–547 (2001). 10.1053/META.2001.22510 11319714 10.1053/meta.2001.22510

[48] A. Brener et al.: Sex differences in body composition in youth with type 1 diabetes and its predictive value in cardiovascular disease risk assessment. Diabetes. Metab. Res. Rev. **39**, (2023). 10.1002/DMRR.3584 10.1002/dmrr.3584 PMC10078230 36269559

[49] G. Rodríguez et al.: Skinfold measurements at birth: sex and anthropometric influence. Arch. Dis. Child. Fetal Neonatal. Ed. **90**, (2005). 10.1136/ADC.2004.060723 10.1136/adc.2004.060723 PMC1721881 15846023

[50] L.M. Maynard et al. Childhood body composition in relation to body mass index. Pediatrics **107**, 344–350 (2001). 10.1542/PEDS.107.2.344 11158468 10.1542/peds.107.2.344

[51] Y.F. Pei et al. The genetic architecture of appendicular lean mass characterized by association analysis in the UK Biobank study. Commun. Biol. **3**, 1–13 (2020). 10.1038/s42003-020-01334-0 33097823 10.1038/s42003-020-01334-0 PMC7585446

[52] J.P. Chaput et al. 2020 WHO guidelines on physical activity and sedentary behaviour for children and adolescents aged 5–17 years: summary of the evidence. Int. J. Behav. Nutr. Phys. Act. **17**, 1–9 (2020). 10.1186/S12966-020-01037-Z/TABLES/6 33239009 10.1186/s12966-020-01037-z PMC7691077

[53] A.G. Johansson, Gender difference in growth hormone response in adults. J. Endocrinol. Invest **22**, 58–60 (1999) 10442572

[54] R. Coutant et al. Divergent effect of endogenous and exogenous sex steroids on the insulin-like growth factor I response to growth hormone in short normal adolescents. J. Clin. Endocrinol. Metab. **89**, 6185–6192 (2004). 10.1210/JC.2004-0814 15579776 10.1210/jc.2004-0814

[55] S.S. Ahmad, K. Ahmad, E.J. Lee, Y.H. Lee, I. Choi: Implications of insulin-like growth Factor-1 in skeletal muscle and various diseases. Cells **9**, (2020). 10.3390/CELLS9081773 10.3390/cells9081773 PMC7465464 32722232

[56] M.L. Adamo, R.P. Farrar, Resistance training, and IGF involvement in the maintenance of muscle mass during the aging process. Ageing Res. Rev. **5**, 310–331 (2006). 10.1016/J.ARR.2006.05.001 16949353 10.1016/j.arr.2006.05.001

[57] G. Vitale, G. Pellegrino, M. Vollery, L.J. Hofland, ROLE of IGF-1 system in the modulation of longevity: controversies and new insights from a centenarians’ perspective. Front. Endocrinol. **10**, 27 (2019). 10.3389/FENDO.2019.00027 10.3389/fendo.2019.00027 PMC6367275 30774624

